# Role of Jagged1/STAT3 signalling in platinum‐resistant ovarian cancer

**DOI:** 10.1111/jcmm.14286

**Published:** 2019-04-16

**Authors:** Jiang Yang, Hui Xing, Danhua Lu, Jun Wang, Bingshu Li, Jianming Tang, Fengqin Gu, Li Hong

**Affiliations:** ^1^ Department of Obstetrics and Gynaecology Renmin Hospital of Wuhan University Wuhan P.R. China; ^2^ Department of Obstetrics and Gynaecology Xiangyang Central Hospital Xiangyang P.R. China

**Keywords:** crosstalk, Jagged1, ovarian cancer, platinum resistance, STAT3

## Abstract

Jagged1, the essential ligand of the Notch signalling pathway, is highly expressed in metastatic prostate cancer, and its high expression in breast cancer is linked to poor survival rates. However, the mechanism of Jagged1′s involvement in platinum‐resistant ovarian cancer has not been thoroughly elucidated to date. The purpose of the present study was to investigate the roles of Jagged1 in the platinum resistance of ovarian cancer and its possible mechanisms. Compared with a platinum responsive group of ovarian epithelial cell carcinomas, we found the positive staining intensity of Notch1, Notch2, Jagged1, STAT3 and Epithelial‐mesenchymal transition (EMT) proteins were lower in a platinum‐resistant group. The DDP‐resistant ovarian cancer cell line (C13K) had a higher IC50 of DDP than its parental cell line (OV2008) (*P *<* *0.05) and acquired an EMT phenotype and invasive characteristics. Inhibiting or knockdown of Jagged1 expression could not only reduce its capacity of migration and invasion but also reverse EMT and down‐regulate the expression of serine 727‐phosphorylated STAT3 (pS727) at the protein level but not total STAT3 or tyrosine 705‐phosphorylated STAT3 (pY705) in C13K cells. Furthermore, it was found that crosstalk between the Jagged1/Notch and JAK/STAT3 signalling pathways were involved in Jagged1‐promoting EMT in C13K cells. Experiments in vivo showed a reduced micrometastatic tumour burden in the lung, liver and spleen of mice implanted with C13K cells with knocked‐down Jagged1 compared with mice implanted with control cells. All of these results demonstrate that Jagged1 can crosstalk with the JAK/STAT3 pathway, and they all cooperate to promote the aberrant occurrence of EMT, further reinforcing the abilities of invasion and migration of platinum‐resistant ovarian cancer in vivo and in vitro.

## 1 INTRODUCTION

Epithelial‐mesenchymal transition (EMT), a process by which epithelial cells lose their cell polarity and gain mesenchymal properties,^1^ enable cells to down‐regulate the expression of cell adhesion molecules, such as E‐cadherin, and up‐regulate the expression of mesenchymal markers, such as N‐cadherin, to adopt migratory and invasive behaviours.^2^ EMT is not only an essential process in normal embryonic development, and increasing numbers of studies have suggested that EMT is also a significant mechanism involved in the progression of various cancers.^3^, ^4^, ^5^ Furthermore, the mesenchymal stem cell properties endowed by EMT have been regarded as a key step in cancers, including ovarian cancer, acquiring metastatic properties and chemoresistance.^3^, ^5^, ^6^, ^7^

Epithelial ovarian cancer is the most lethal gynaecologic malignancy, although it only accounts for approximately 5% of all female‐specific cancers.^8^ Although ovarian cancer is sensitive to platinum‐based systemic chemotherapy treatment, its clinical course suggests that a population of neoplastic cells has either an inherent or acquired resistance to chemotherapy that enables survival during initial therapy and ultimately leads to recurrence.^9^, ^10^ Over the past several decades, the overall survival (OS) and progression‐free survival (PFS) of ovarian cancer patients have only shown modest improvements. Therefore, a better understanding of the cellular pathways involved in the malignant characteristics of cisplatin‐resistant ovarian tumours is imperative for the improvement of therapeutic approaches.

The Notch pathway, an essential signalling pathway in cell growth and differentiation during embryonic development, has been reported to participate in multiple malignancies, including ovarian cancer.^11^, ^12^ Furthermore, this pathway is especially critical in maintaining the subpopulation of cancer cells with stem cell properties and conferring resistance to chemotherapies.^13^, ^14^ In turn, the stem cell properties further promote the biological behaviour of cancer cells, such as unlimited proliferation, invasion and migration. The Notch signalling pathway is composed of receptors (Notch1, 2, 3 and 4), ligands (Jagged1, 2 and Delta‐like ligand (DLL) 1, 3 and 4) and intracellular domains (ICD). Most researchers have focused on the effects of inhibiting the Notch receptor and its downstream signalling; however, the Notch signalling pathway that regulates multiple cellular processes can be either oncogenic or tumour suppressing depending on the cancer types,^15^, ^16^ and the biological functions of its ligands have not been thoroughly characterized to date. Recently, studies have reported that reduced expression or dysregulation of DLL4 is a key mechanism for Notch‐mediated predisposition to carcinogenesis and tumourigenesis in Li‐Fraumeni syndrome (LFS)^17^ and the DLL1 and DLL4 ligands are required for maintenance of intestinal progenitor and stem cells (SCs) and are involved in EMT.^18^, ^19^ Jagged 1 is highly expressed in metastatic prostate cancer and high expression of Jagged1 in breast cancer is linked to poor survival rates.^20^, ^21^ Nevertheless, the mechanism of Jagged1 in cisplatin‐resistant ovarian cancer is still unclear.

In stem cells and breast cancer, the activity of the Notch signalling pathway is critical for activation of signal transducer and activator of transcription 3 (STAT3).^22^, ^23^ STAT3 activation is not only associated with increases in malignant cancer behaviours (uncontrolled growth, migration, invasion and therapeutic resistance),^24^, ^25^ it may also exert a critical influence on establishing cell polarity during directed cancer cell progression, processes significant for EMT programmes.^26^

In this study, we demonstrated that activation of Jagged1 induces EMT in cisplatin‐resistant ovarian cancer cells. In addition, our results suggest that Jagged1/Notch and JAK/STAT3 signalling form a positive regulatory loop and cooperatively regulates EMT and promote cisplatin‐resistant ovarian cancer cell invasion and migration.

## 2 MATERIALS AND METHODS

### 2.1 Tissue samples

This study was approved by the ethics committee of Renmin Hospital of Wuhan University (Wuhan, China), and each participant provided written informed consent. A total of 42 patients were enrolled from the Department of Obstetrics and Gynaecology, Renmin Hospital of Wuhan University between 2016 and 2017. All of the eligible patients fulfilled the following criteria: (a) Platinum chemotherapy‐sensitive cases: complete remission (CR) was achieved after adjuvant chemotherapy, and the interval between the last chemotherapy treatment and recurrence was greater than or equal to 6 months. (b) Platinum‐resistant cases: complete remission was achieved after adjuvant chemotherapy, and the recurrence time was <6 months after the last chemotherapy treatment. (c) Refractory cases of platinum‐based chemotherapy: the best response to platinum‐based chemotherapy during disease progression (PD) or after at least six courses of platinum‐based chemotherapy was a partial remission (PR) or disease stability (SD). Groups (b) and (c) are collectively referred to as platinum‐based chemotherapy‐resistant cases in this study. The study group consisted of 33 platinum chemotherapy‐sensitive cases and nine platinum‐based chemotherapy‐resistant cases. All sample tissue blocks were fixed for 12‐24 hours at room temperature in a 4% neutrally buffered paraformaldehyde solution, dehydrated, embedded in paraffin, and then sliced into 4‐μm sections for immunohistochemical staining.

### 2.2 Cell lines and culture

A cisplatin‐sensitive human epithelial ovarian cancer cell line (OV2008) and its resistant variant (C13K) were gifts from professor Ma Ding (Cancer Biology Research Center, Huazhong University of Science and Technology, China). Cells were maintained in RPMI‐1640 (Jenom, Hangzhou, China) complete medium with 100 U/mL penicillin/streptomycin (Beyotime Institute of Biotechnology, Haimen, China) and 10% foetal bovine serum (FBS; Gibco‐BRL, Invitrogen Life Technologies) at 37°C in a humidified atmosphere containing 5% CO_2_.

### 2.3 RNA interference

Small interfering (siRNA) sequences directed against Jagged1 and Jagged2 were designed and synthesized by the Guangzhou RiboBio Company (Guangzhou, China). The sequences for Jagged1 and Jagged2 were: 5′‐GAAGAATGTTTCCGCTGAA‐3′ and 5′‐GCAAAGAAGCCGUGUGUAA‐3′, respectively. The empty vector was used as the control. For transfection, the cells were resuspended at a density of 5 × 10^4^ cells/mL and seeded in six‐well plates. When the cells were 60%‐80% confluent, they were transfected using HiPerfect (QIAGEN, Duesseldorf, Germany) according to the manufacturer's instructions. The transfected cells were named C13K/si‐Jagged1, C13K/si‐Jagged2 or C13K/si‐NC depending on the treatment. To explore the effects of Jagged1 knockdown in ovarian epithelial cancer (OEC) tumourigenesis in vivo, small interfering (siRNA) sequences were designed as shRNA and were packaged with lentiviral vectors. Cells were grown to 30%‐40% confluence and incubated with the constructed lentiviral vectors for 8 hours in growth medium containing10 μg/mL of polybrene (Santa Cruz). Three days after infection, the medium was changed to fresh RPMI 1640 containing 3 μg/mL puromycin (Santa Cruz). Puromycin‐resistant colonies were used for subsequent studies in vivo.

### 2.4 Cell viability and proliferation assay

Cell viability and IC50 values (drug concentration causing 50% inhibition of cell growth) were measured by the CCK‐8 assay (Beyotime Institute of Biotechnology, Haimen, China). Briefly, cells were suspended in RPMI1640 medium at a density of 5 × 10^4^ cells/mL and seeded into 96‐well plates and incubated for 24 hours at 37°C. The CCK‐8 assay was performed before transfection and then at 24, 48, 72 and 96 hours following transfection for the control groups. For the treatment groups, following removal of the spent culture medium, 100 μL of prepared medium containing various concentrations of DAPT (an inhibitor of the γ‐secretase complex, and it can indirectly inhibit the Notch pathway) (0, 2.5, 5, 10, 20 or 40 μmol/L), an inhibitor of the γ‐secretase complex, and it can indirectly inhibit the Notch pathway, was added. Each treatment was conducted in triplicate. Following incubation at 37°C for 24 hours, 10 μL of CCK‐8 solution was added to each well and the cells were incubated for 1‐2 hours. The absorbance of the wells was then measured at 450 nm using a microplate spectrophotometer (Victor3 1420 Multilable Counter; Perkin Elmer, Inc., Waltham, MA, USA). The viability of the treated group was expressed as a percentage of the untreated control group, which was designated as 100%. Cell growth curves were plotted as follows: Cellular growth (%) = OD450 of experimental well)/OD450 of control well.

### 2.5 Three‐dimensional (3D) spheroid formation

A total of 5 × 10^3^ cells was seeded on Matrigel (50 μL/cm^2^, cat. 356234; Corning Incorporated, NY, USA) coated 24‐well plates, and the media were refreshed every 2‐3 days. The cell forming 3D spherical structure was photographed (×200 magnifications) at 2‐day intervals for 2 weeks.

### 2.6 Wound‐healing assay

Cells were seeded and grown in six‐well plates to complete confluence. A wound injury was made with the yellow tip of a sterile micropipette, and the detached cells were removed by washing with phosphate buffer solution (PBS). Cells were then incubated with the prepared medium containing various concentrations of DAPT (0, 2.5, 5, 10, 20 or 40 μmol/L) and allowed to migrate for up to 24 hours. Images were taken at 4 hours intervals by inversion microscope (BX51; Olympus Corporation, Tokyo, Japan). Relative wound density was calculated by ImageJ Software 1.51 (https://imagej.nih.gov/ij/) as the metric to quantify cell migration.^27^

### 2.7 Cell migration and invasion assays

Migration and invasion assays were performed by using 24‐well Transwell plates (Corning Incorporated) as instructed by the manufacturer. Briefly, cells (5 × 10^4^ cells/well for the migration assay and 1 × 10^5^ cells/well for the invasion assay) were plated in serum‐free medium in the upper chambers and in RPMI 1640 containing various concentrations of DAPT (0, 2.5, 5, 10, 20 or 40 μmol/L) with 10% FBS placed in the bottom wells and incubated at 37°C for 24 hours. The migrated or invaded cells were fixed with 4% paraformaldehyde followed by staining with 1% crystal violet. Three random microscopic fields (×100) of the chamber were photographed and counted.

### 2.8 Bimolecular fluorescence complementation assay

The slides of C13K cells were fixed with 4% formaldehyde for 15 minutes and after washing with PBS, the cells were incubated with 0.1% Triton X‐100 for 5 minutes at room temperature. The slides were blocked with 2% goat serum, followed by incubation overnight with the primary antibodies (a mixture of mouse polyclonal anti‐STAT3 antibody and rabbit polyclonal anti‐Jagged1 antibody) at 4°C. The next day, the cells were incubated with the secondary antibody mixture containing fluorescent‐labelled goat antimouse polyclonal IgG secondary antibody (CY3, 1:100, BA1032, BOSTER Biological Technology Co. Ltd., Wuhan, China) and goat anti‐rabbit polyclonal IgG secondary antibody (FITC, 1:50; BA1105, BOSTER Biological Technology Co. Ltd.) at room temperature for 60 minutes, and the nuclei were stained using Diamidine phenyl indole (DAPI) at room temperature for 10 minutes. The cells were observed under a fluorescence microscope (Olympus Corporation).

### 2.9 Western blot analysis

The cell lysates were electrophoresed on sodium dodecyl sulphate polyacrylamide gel electrophoresis (SDS‐PAGE) and transferred onto polyvinylidene fluoride (PVDF) membranes (EMD Millipore, Billerica, MA, USA). The membranes were blocked with 5% non‐fat milk solution before primary antibody incubation overnight at 4°C. After washing, the membrane was incubated with horseradish peroxidase‐conjugated secondary antibodies (1:10 000 dilution; LICOR Biosciences, Lincoln, NE, USA) for 1 hour. Finally, the staining intensity was visualized and quantified by an Odyssey imaging system (LICOR Biosciences, Lincoln, NE, USA). Experiments were performed in triplicate. The following primary antibodies were used: mouse polyclonal anti‐STAT3 antibody (1:1000; cat. no. 9139), mouse polyclonal anti‐phospho‐STAT3 (Tyr705;1:2000; cat. no. 9145), rabbit polyclonal anti‐phospho‐STAT3 (Ser727; 1:1000; cat. no. 94994), mouse polyclonal anti‐E‐cadherin antibody (1:1000; cat. no.14472), mouse polyclonal anti‐N‐cadherin antibody (1:1000; cat. no.13116), rabbit monoclonal anti‐vimentin antibody (1:1000; cat. no. 5741) and rabbit monoclonal anti‐cleaved Notch1 antibody (1:1000; cat. no. 4147), all obtained from Cell Signaling Technology, Inc. (Danvers, MA, USA); rabbit polyclonal anti‐Jagged1 antibody (1:500; cat. no. ab7771), rabbit polyclonal anti‐Jagged2 antibody (1:2000; cat. no. ab109627), rabbit monoclonal anti‐Notch1 antibody (1:500; cat. no. ab8925), rabbit monoclonal anti‐Notch2 antibody (1:500; cat. no. ab8926) and rabbit monoclonal anti‐Twist1 antibody (1:1000; cat. no. ab50581) were obtained from Abcam, Inc. (Cambridge, USA). Rabbit monoclonal anti‐cleaved Notch2 antibody (1:100; cat. no. 40517) was obtained from Signalway Antibody LLC (Maryland, USA). An anti‐β‐actin antibody (1:1000; cat. no. 4970; Cell Signaling Technology, Inc.) served as an endogenous reference.

### 2.10 Reverse transcription‐polymerase chain reaction (RT‐PCR)

Total RNA from DMSO‐treated control and PEITC‐treated cells was isolated using RNeasy kit (Qiagen). First‐strand cDNA was synthesized using Superscript reverse transcriptase (Invitrogen‐Life Technologies) with oligo (dT)20 primer; semi‐quantitative and real‐time PCR were performed (Primers in Table S1).

### 2.11 Immunoprecipitation

Cells were solubilized in lysis buffer. An equal amount of each protein lysate was incubated with anti‐STAT3 or anti‐Jagged1 antibody overnight at 4°C, followed by incubation with 10 μL of protein A‐sepharose beads for 2 hours. The immune complexes were analysed by Western blot analysis with anti‐Jagged1 or anti‐STAT3 antibody.

### 2.12 Immunohistochemistry

An ElivisionTM super HRP IHC kit (cat. no. Kit‐9921; Fuzhou Maxim Biotechnology Development Co., Ltd.) was used for immunohistochemistry. The paraffin‐embedded sections were deparaffinized, rehydrated and antigen retrieval was performed by heat mediation in 0.01 M sodium citrate, pH 6.0. The sections were incubated overnight with mouse polyclonal anti‐STAT3 antibody (1:500; Cell Signaling Technology, Inc.), rabbit polyclonal anti‐Jagged1 antibody (1:200; Abcam, Inc.), rabbit monoclonal anti‐Notch1 antibody (1:150; Cell Signaling Technology, Inc.), or rabbit monoclonal anti‐Notch2 antibody (1:100; Cell Signaling Technology, Inc.) at 4°C following the instructions on the product datasheets for the primary antibodies. Following incubation with streptavidin peroxidase (Maxim Biotechnology Development Co., Ltd.) for 10 minutes at room temperature, secondary antibodies were added for 30 minutes at 37°C. The immune reaction was then visualized using 3,3'‐diaminobenzidine (DAB) (Fuzhou Maxim Biotechnology Development Co., Ltd., Fuzhou, China). The expression intensity was evaluated by a semi‐quantitative system to calculate the percentage of positive neoplastic cells: 0 points, no positive cells; 1 point, 1% to 25%; 2 points, 26% to 50%; 3 points, 50% to 75%; 4 points, >75%. Then, ≤2 points were judged as negative for expression, 2 points, were judged as positive expression.

### 2.13 Xenograft tumourigenesis in nude mice

Athymic nude mice (BALB/c nu/nu) (female, 5‐week old) were purchased from Beijing Vital River Laboratory Animal Technology Cooperation (Beijing, China) and were acclimated for 7 days in the laboratory before experimentation. To establish the capability of invasiveness and migration, 1 × 107 Jagged1 knockdown, negative control and C13K cells were injected into the tail vein of the mice (n = 4/group). Body weight was measured once a week. On the day of harvest the lung, liver and spleen were analysed by the haematoxylin and eosin staining method. All animal studies were approved by the ethics committee of the Renmin Hospital of Wuhan University.

### 2.14 Statistical analysis

Statistical analysis was performed with the statistical Package for Social Science (SPSS Release 22.0; SPSS Inc., Chicago, IL, USA). The differences observed between the control and treated groups for cell proliferation, viability, cell migration and invasion ability, the mRNA and proteins expression were analysed using either one‐way ANOVA or unpaired Student's *t* tests (two‐tailed). Chi‐squared test was used to analyse the protein expression intensity between platinum‐resistant group and platinum responsive group. The results are expressed as the mean ± standard deviation from triplicate experiments and a value of *P* < 0.05 was considered to be statistically significant.

## 3 RESULTS

### 3.1 Notch pathway in platinum‐resistant ovarian cancer is important for the malignant phenotype

**Figure 1:**
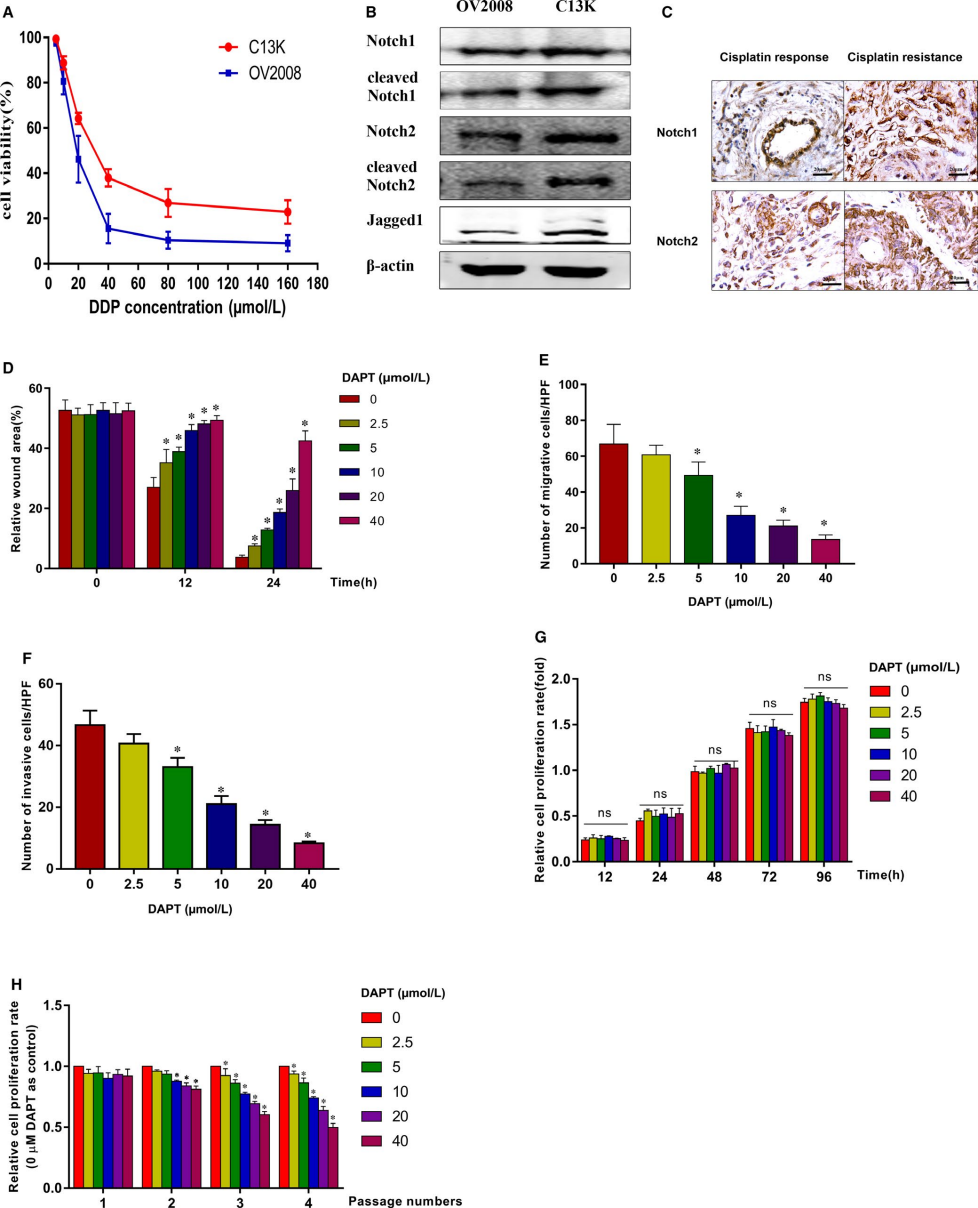
The Notch pathway in platinum‐resistant ovarian cancer is important for cell malignant phenotype. A, The cytotoxic effect of cisplatin (IC50) on OV2008 and C13K cells were examined by CCK‐8 assay. B, The protein expression levels of Notch1/2 and cleaved Notch1/2 in OV2008 and C13K cells were determined by Western Blot. C, Immunohistochemistry analyses of Notch1 and Notch2 were performed in platinum‐resistant group and platinum responsive group, as shown in representative images (×400 magnification). D, Wound healing assay was analysed the migratory ability of C13K cells treated by a wide concentration range of DAPT (0, 2.5, 5,10, 20 and 40 μmol/L). (E and F) Transwell migration and invasion assay were performed to confirm the migratory and invasive abilities of C13K cells exposed by a wide concentration range of DAPT. G and H, CCK‐8 proliferation assay was examined the proliferative ability of C13K cells treated by a wide concentration range of DAPT for different time. (*P < 0.05)

In this study, we first examined the cytotoxic effect of cisplatin on OV2008 and C13K cells by using a CCK‐8 assay. The IC50 value was used to represent the level of cytotoxicity. The IC50 values for the OV2008 and C13K cells were 23.11 ± 0.97 μmol/L and 39.43 ± 1.19 μmol/L, respectively (Figure 1A), which suggested that the C13K cells were more resistant to cisplatin‐induced cytotoxicity compared with the OV2008 cells. To investigate whether the Notch pathway was involved in the cisplatin resistance of ovarian cancer, we first examined the protein expression levels of this pathway. Western blot analysis determined that the expression levels of Notch1/2 and cleaved Notch1/2 in C13K cells were significantly higher than in OV2008 cells. Moreover, the Jagged1 protein level and mRNA level were also highly expressed in C13K cells (Figure 1B and Figure S1). To confirm whether these findings were consistent with that in actual human tumours, the relative genes' protein expression levels were examined by IHC of the tissues in the platinum‐resistant group and platinum responsive group. The results showed that Notch1 and Notch2 were expressed in all tumour samples from the platinum‐resistant group and most of the tumour samples from the platinum responsive group, and in addition, Notch1 and Notch2 positive staining intensities were higher in the platinum‐resistant group than in the platinum responsive group (Figure 1C and Table S2).

To illuminate the role of the Notch pathway in C13K cells, DAPT was applied and its effects on cell proliferation and migratory ability were examined. First, we examined the effect of DAPT on cell migration and invasion abilities. Wound healing assays showed that the wound density in C13K cells was significantly higher after DAPT exposure (Figure 1D and Figure S2). Moreover, the Transwell migration assay confirmed that DAPT treatment greatly suppressed the migratory ability of the C13K cells (Figure 1E and Figure S3) and their invasive ability (Figure 1F and Figure S4). Therefore, these findings implicated the Notch pathway as playing an important role in the migration and invasive abilities of cisplatin‐resistant ovarian cancer cells. We also checked whether DAPT could inhibit the proliferation of the C13K cells. However, we found that the cell proliferation rates up to 72 hours after DAPT treatment showed no significant change (Figure 1G). In case a continuous exposure to DAPT was not sufficient, we also maintained continuous exposure of C13K cells to different concentrations of DAPT for one to four passages and we observed that the cell proliferation was gradually decreased following passage 2 in a dose‐dependent manner (Figure 1H). These results suggest that the Notch pathway in cisplatin‐resistant ovarian cancer is significant in increasing the cell malignant phenotype.

### 3.2 Notch pathway is involved in EMT progression in cisplatin‐resistant ovarian cancer cells

**Figure 2:**
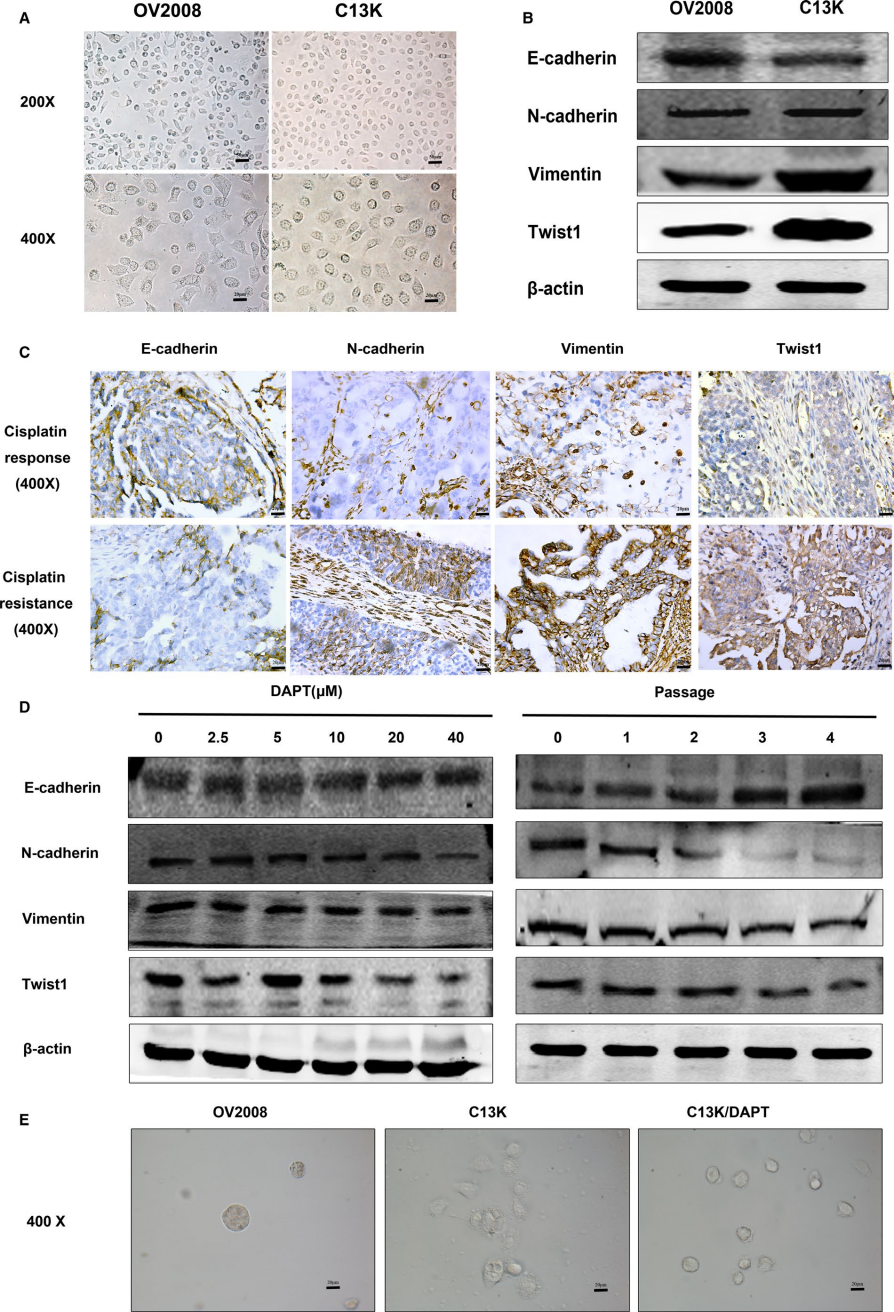
The Notch pathway is involved in epithelial‐mesenchymal transition (EMT) progression in cisplatin‐resistant ovarian cancer cells. A, Optical microscope was designed to observe the morphology of C13K cells and OV2008 cells. B, The protein expression levels of EMT relative genes were determined by Western Blot. C, Immunohistochemistry analyses of EMT relative proteins were performed in platinum‐resistant group and platinum responsive group, as shown in representative images (×400 magnification). D, Western blot assay examined the expression of EMT relative proteins of C13K cells treated by a wide concentration range of DAPT for different time. E, The aggressive phenotype of OV2008 cells and C13K cells in the presence or absence of DAPT (10 μmol/L) when cultured in three‐dimensional matrigel

First, we found that the morphology of the C13K cells showed changes to a shuttle and stem‐like shape (Figure 2A and Figure S5), which are consistent with morphological EMT features. Western blot analyses revealed that the expression of the epithelial adhesion protein E‐cadherin was lower, while the mesenchymal marker proteins N‐cadherin and vimentin as well as the EMT key modulator Twist1 were up‐regulated in C13K cells (Figure 2B). Furthermore, IHC assay showed that the positive staining intensity of the EMT related mesenchymal proteins (N‐cadherin, vimentin and Twist1) were higher in the platinum‐resistant group than in the platinum responsive group (Figure 2C and Tables S3 and S4). These results suggested that EMT is a critical phenotype change in platinum‐resistant ovarian cancer.

To further explore the role of Notch related to EMT signalling in C13K cells, exposure to a wide concentration range of DAPT was used. The protein expression of Twist1 was inhibited when the C13K cells were treated with 10 μmol/L DAPT for 48 hours, but the E‐cadherin, N‐cadherin and vimentin expression levels were not obviously changed (Figure 2D). The reason for this result was either not a long enough exposure time or the Notch inhibitor had no effects on EMT. Dramatically, when we examined the protein levels after continuous exposure of C13K cells to 10 μmol/L DAPT for one to four passages, we found that E‐cadherin expression recovered and the N‐cadherin and vimentin protein levels were down‐regulated in a dose and time‐dependent manner (Figure 2D). Furthermore, we employed 3D culture to examine the characteristics of cancer stem cells (CSCs), which was an important functional change endowed by EMT. When C13K cells were cultured for 2 weeks, we found that they displayed an aggressive phenotype, showing highly disorganized cell clusters lacking basal polarity, while OV2008 cells and C13K cells treated with DAPT showed a lower aggressive ability and more organized spheroid structures (Figure 2E and Figure S6). These results suggested that the Notch pathway could regulate the occurrence of EMT in cisplatin‐resistant ovarian cancer cells.

### 3.3 Jagged1 plays a critical role in EMT signalling in C13K cells

**Figure 3:**
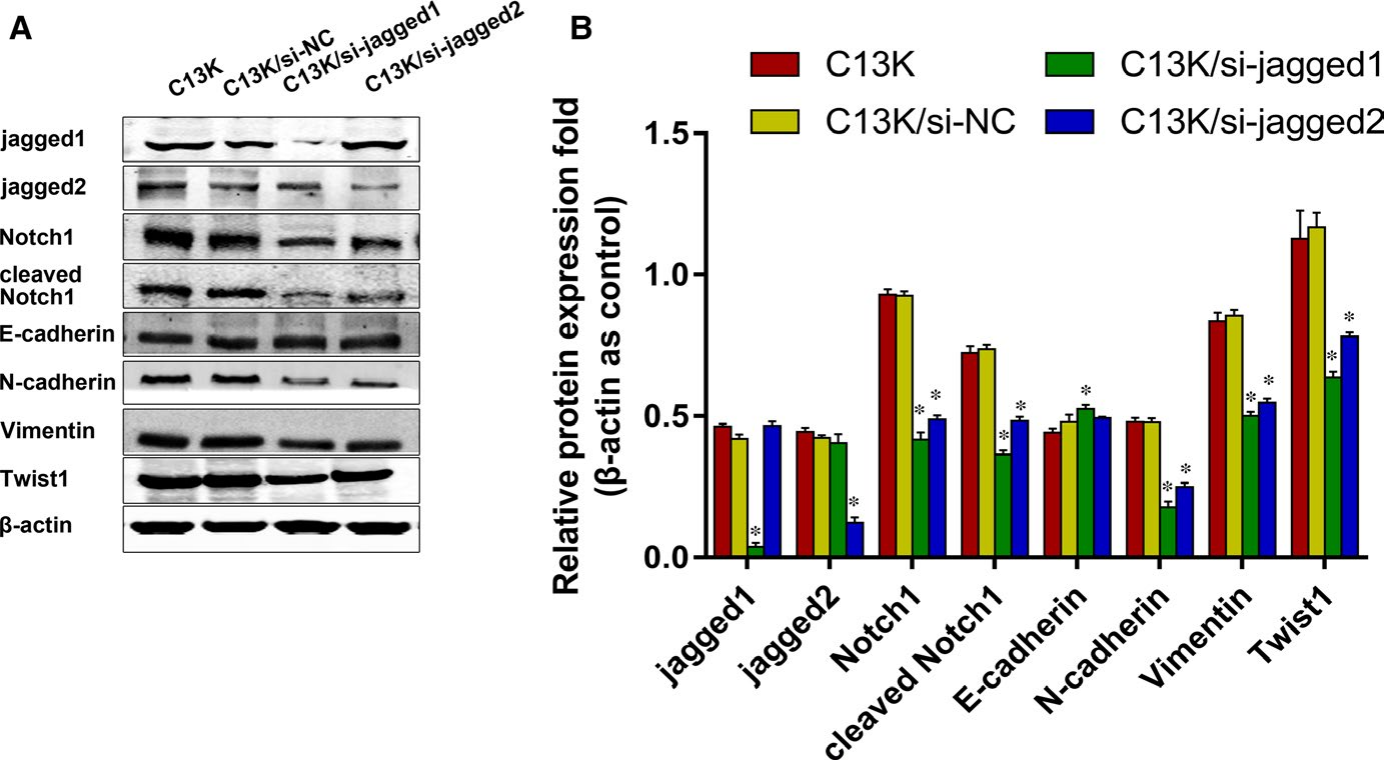
Jagged1 plays a critical role in EMT signalling in C13K cells. A, Western blot assay examined the expression of Notch1, cleaved Notch1 and EMT relative proteins of C13K cells after transfection with Jagged1 and Jagged2 siRNA. B, The quantitative analysis of Fig. 3A. (*P < 0.05)

Jagged1 is one the most important ligands of the Notch pathway, and studies have demonstrated it has a role in regulating EMT of cancer.^28^ Jagged2, a homologue of Jagged1, also plays an important role in EMT of lung adenocarcinoma.^29^ To investigate whether Jagged1 and/or Jagged2 are critical for EMT progression in C13K cells, the cells were transfected with control, Jagged1 and Jagged2 siRNA. Figure 3A and B show strong suppression of Notch1 and cleaved Notch1 after transfection with each target siRNA. Western blot assays showed that the expression of N‐cadherin and vimentin, mesenchymal markers of EMT, were reduced by Jagged1 and Jagged2 knockdown in C13K cells, and the expression of these proteins was lower in C13K/si‐Jagged2 cells compared with C13K/si‐Jagged1 cells. A similar result was found for Twist1, a key transcription regulator of EMT. Although the protein expression of E‐cadherin was not completely restored, a tendency towards reversion was observed following Jagged1 knockdown (Figure 3). These results suggest that Jagged1 and Jagged2 are involved in the Notch pathway, and Jagged1 plays a more important role in EMT signalling in C13K cells.

### 3.4 Crosstalk of Jagged1/STAT3, not Jagged2/STAT3, is important for EMT in C13K cells

**Figure 4:**
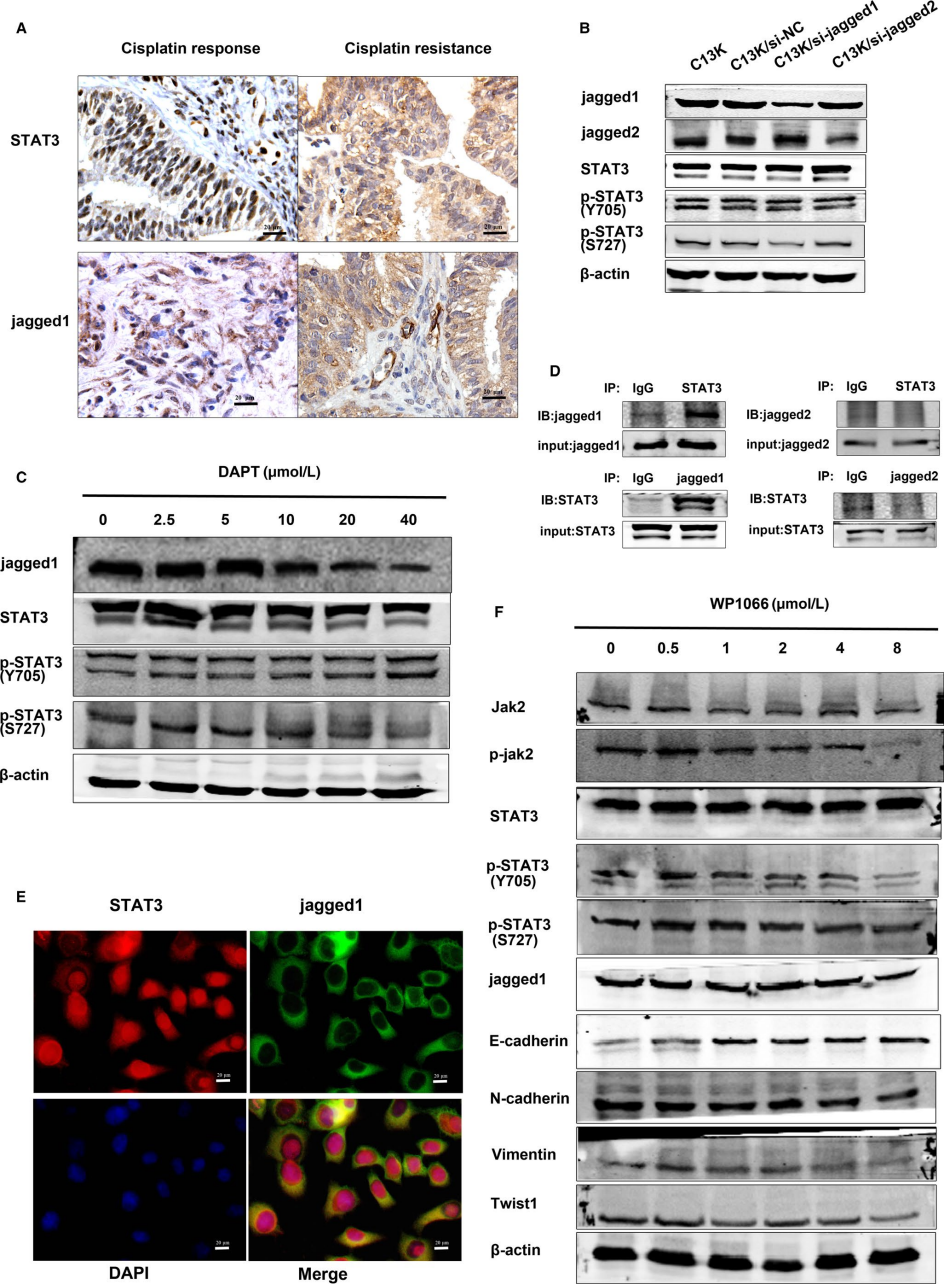
The crosstalk of Jagged1/STAT3, not Jagged2/STAT3, is important for epithelial‐mesenchymal transition (EMT) in C13K cells. A, Immunohistochemistry analyses of STAT3 and Jagged1 proteins were performed in platinum‐resistant group and platinum responsive group, as shown in representative images (×400 magnification). B, Western blot assay examined the expression of Jagged1, Jagged2 and JAK/STAT3 pathway relative proteins of C13K cells after transfection with Jagged1 and Jagged2 siRNA. C, The proteins expression of Jagged1, Jagged2 and JAK/STAT3 pathway relative proteins of C13K cells treated by a wide concentration range of DAPT (0, 2.5, 5,10, 20 and 40 μmol/L). D, The physical relationship of Jagged1 and STAT3 was analysed by Co‐IP assay. E, The distribution of Jagged1 and STAT3 in C13K cells was examined by co‐immunofluorescent staining and confocal microscopy imaging. F, The proteins expression of Jagged1, JAK/STAT3 pathway and epithelial‐mesenchymal transition (EMT) relative proteins of C13K cells treated by a wide concentration range of WP1066 (0, 0.5 1, 2, 4 and 8 μmol/L)

What could be causing the stronger ability of Jagged1 in regulating EMT in C13K cells? Recently, a study reported that Notch4/STAT3 crosstalk is important for EMT in breast cancer,^23^ and IHC assays showed that Jagged1 and STAT3 protein were both expressed in the tissues of the cisplatin‐resistant group and the cisplatin responsive group. The STAT3 protein was mainly localized to the cell nucleus with some molecules localized to the cytoplasm and cytomembrane. Jagged1 protein was mainly localized to the cytomembrane and only a few molecules were localized to the cytoplasm and cell nucleus, and the staining intensity of both proteins were stronger in the cisplatin‐resistant group (Figure 4A and Table S5). Therefore, we examined the protein level of STAT3 in C13K cells transfected with Jagged1 and Jagged2 siRNA. Western blot assay showed that the levels of total STAT3 and tyrosine 705‐phosphorylated STAT3 (pY705) were not obviously altered in C13K/si‐Jagged1 and C13K/si‐Jagged2 cells, while the serine 727‐phosphorylated STAT3 (pS727) protein level was significantly reduced by knocking down Jagged1 in C13K cells but not by knocking down Jagged2 (Figure 4B and Figure S7). In addition, we observed similar results with C13K/si‐Jagged1 cells and C13K cells treated with DAPT, with a reduction in pS727 protein in a dose‐dependent manner (Figure 4C).

To further explore the physical association between Jagged1 and STAT3, total cell lysates from C13K cells were immunoprecipitated using an anti‐STAT3 antibody. The co‐IP assay showed that STAT3 physically interacts with Jagged1 but not Jagged2 (Figure 4D). We further performed a bimolecular fluorescence complementation assay in C13K cells and we detected that both STAT3 and Jagged1 are localized to the cytoplasm and cytomembrane, and the interaction of these proteins was confirmed by co‐immunofluorescent staining and confocal microscopy imaging (Figure 4E). These results suggest that Jagged1 could regulate the protein expression of STAT3 and Jagged1/STAT3 crosstalk may play an important role for EMT in C13K cells. Interestingly, Yang et al^30^ have reported that acquisition of trastuzumab resistance is associated with the formation of the EMT/CSC phenotype and transition of survival signalling through activating an IL‐6/STAT3/Jagged‐1/Notch positive feedback signalling loop in gastric cancer cells crosstalk. Therefore, we exposed C13K cells to a wide concentration range of WP1066, an inhibitor of the JAK/STAT3 pathway. Western blot assay showed that the Jagged1 level was not altered obviously and the levels of N‐cadherin and vimentin, mesenchymal markers of EMT, were inhibited by WP1066 in a dose‐dependent manner (Figure 4F). Based on these results, we could conclude that Jagged1/STAT3 crosstalk is a critical mechanism for EMT in cisplatin‐resistant ovarian cancer.

### 3.5 Jagged1 knockdown impairs tumour growth and invasion in mouse xenograft models

**Figure 5:**
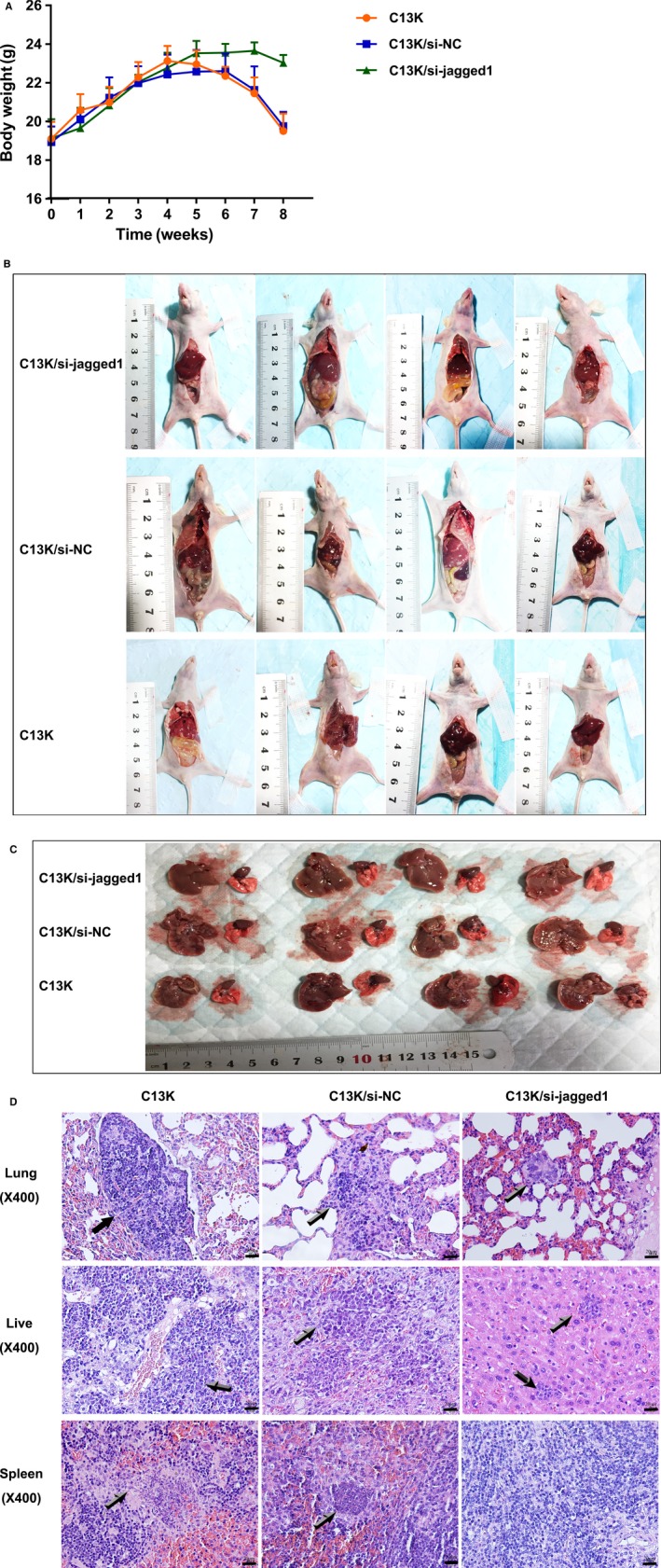
Knock‐down Jagged1 impairs tumour growth and invasion in mouse xenograft models. A, The weight of each mice bearing C13K/si‐Jagged1 cells, C13K cells and C13K/si‐NC cells were measured once a week. B, C13K/si‐Jagged1 cells, C13K cells and C13K/si‐NC cells were injected into tail veins of the nude mice, respectively. 8 weeks following tumour cell implantation, the mice were photographed after harvest. C, The tissue sample (lung and liver) each group were photographed after harvest. D, Representative images of haematoxylin and eosin‐stained lung, liver and spleen sections from each group

To explore the effects of Jagged1 knockdown on OEC tumourigenesis in vivo, 1 × 10^7^ OEC cells were injected into the tail veins of each nude mouse. Mice were divided into three groups: mice bearing control C13K cells (group 1), mice bearing C13K/si‐NC cells (group 2) and mice bearing C13K/si‐Jagged1 cells (group 3), and the weight of each mouse was measured once a week. As shown in Figure 5A, there were no obvious differences in body weight amongst these three groups before 4 weeks after injection. However, the first group and the second group began losing weight after the fourth week and developed cachexia at 7 to 8 weeks, while the third group did not show weight loss until the seventh week. This suggests that knockdown of Jagged1 dramatically impaired the tumourigenic growth of C13K cells. At harvest (Figure 5B‐D), mice implanted with C13K/sh‐Jagged1 cells showed no tumour formation in the spleen and only 50 percent (2/4) had liver metastases. In contrast, mice implanted with C13K and C13K/si‐NC cells exhibited aggressive tumour formation in the spleen (3/4) and increased susceptibility to macroscopic metastases in the liver (4/4), and even worse, large areas of necrosis occurred in the spleen and liver, and a large number of metastatic carcinomas were found in the lung in group 1 and 2. Although there were some macroscopic metastases in the lung in group 3, the size of the metastatic carcinoma was one‐fifth that of group 2 and 3. These results confirmed the inhibitory effect of Jagged1 knockdown on cisplatin‐resistant ovarian cancer growth.

## 4 DISCUSSION

Notch signalling regulates a diverse array of cell fate decisions in multiple tissues during both development and homeostasis, including lineage commitment, differentiation, cell cycle progression, and maintenance and self‐renewal of stem cells.^31^, ^32^, ^33^ More surprising, the impact of Notch signalling is exquisitely context dependent, such as showing oncogenic and tumour‐suppressive functions in different cancer types.^15^, ^16^ Application of the DMBA‐TPA mouse model of cutaneous chemical carcinogenesis system to Notch1^−/−^ skin results in a dramatic increase in tumour burden with respect to both the number of benign papillomas and the proportion that progress to squamous cell carcinomas (SCCs).^34^ Subsequently, a body of research has proven that the development and progression of SCCs in various epithelial tissues is strongly associated with loss of Notch signalling.^33^ However, the Notch pathway is postulated to play an oncogenic role in brain cancer, breast cancer, non‐small cell lung cancer (NSCLC) and ovarian cancer.^33^ In ovarian cancer patients, Notch receptors (Notch1‐4) mRNA high expression is not only significantly associated with poor PFS,^35^ but increased protein expression of Notch1 also correlates with poor overall survival (OS).^36^ What's more, Jagged1 was found to be the primary Notch ligand expressed in ovarian cancer cells compared with Jagged2 and DLL1, 3 and 4,^37^ and its increased expression correlates with reduced OS and PFS in women with advanced breast cancer,^20^, ^38^ as well as those with tamoxifen (TAM) resistance.^23^ In this study, we observed increased Jagged1, Notch1/2 and their ICD expression in C13K cells compared with its parent line, OV2008 cells. In human tumour tissues, cisplatin‐resistant ovarian cancer group also showed high protein levels of Jagged1 and Notch1/2 compared with the cisplatin responsive group.

Although the primary treatment for ovarian cancer according to the National Comprehensive Cancer Network (NCCN) is appropriate surgical staging and cytoreduction followed by systemic chemotherapy, the major obstacle for patients to benefit from the chemotherapy is ether intrinsic or acquired resistance to chemotherapy. The latter hypothesis is consistent with what has become known as EMT. The EMT process is commonly believed to have contributed to the establishment of migratory and invasive mesenchymal phenotypes, and resistance to chemotherapy. In tumours with malignant characteristics, especially chemoresistance, it has been reported they contain a small proportion of CSCs, and CSCs have been examined for molecular pathways and markers that could be targeted for therapeutic purposes. In addition, the formation of CSCs is always regulated by EMT. Recent studies have shown that overexpression of the Notch ICD alone results in the loss of E‐cadherin and suppression of Notch signalling abrogated the reduced E‐cadherin expression and increased N‐cadherin, which suggested that the Notch pathway is a critical regulatory mechanism for EMT.^39^ In agreement with this finding, compared with OV2008 cells, EMT key modulator and mesenchymal markers were greatly up‐regulated in C13K cells, and epithelial markers were enormously depleted in this cell type, and highly disorganized cell clusters lacking basal polarity formed under 3D conditions. The cells' migratory and invasive capacities were obviously attenuated in C13K cells by either Notch inhibitors or Jagged1 knockdown. The in vitro study showed similar results. Especially, long‐term culture of C13K cells with DAPT, a Notch pathway inhibitor, eventually led to cell proliferation inhibition and partial reversal of EMT by restoring E‐cadherin expression in C13K cells. What's more, by target genes knockdown, we found Jagged1 plays a more important role in mediating EMT processes in C13K cells than Jagged2. This is consistent with the finding that Jagged1 knockdown cells retained an epithelial morphology and failed to disassemble E‐cadherin adherens junctions and cortical actin bundles.^40^ Furthermore, Choi and Steg et al also demonstrated that Jagged1 is the main Notch ligand in ovarian cancer and silencing it reduced viability and sensitized them to taxane treatment both in vitro and in vivo, where it drastically reduced tumour growth.

There is crosstalk between the Notch pathway and several signalling pathways, such as the TGF‐β/Smad pathway, and this crosstalk modulates the occurrence of EMT that promotes the establishment of migratory and invasive phenotypes.^23^, ^40^, ^41^ STAT3, as one of the most important members of the JAK/STAT3 signalling pathway, exerts a critical influence on establishing cell polarity during directed cancer cells progression.^26^ In response to stimulation, phosphorylation of Tyr 705 on STAT3 stimulates cell differentiation, and phosphorylation of a serine at position 727 is correlated with survival.^22^, ^42^ Quyen et al^23^ have reported that Notch4 could crosstalk with STAT3 and further regulate the progression of EMT in tamoxifen‐resistant human breast cancer, and they also showed tamoxifen‐resistant human breast cancer cells exhibited enhanced phosphorylation of STAT3 at the tyrosine 705 residue. What's more, Androutsellis et al^22^ found that Jagged1 could induce phosphorylation of STAT3 on Ser 727 in a dose‐ and time‐dependent manner in foetal neural stem cells. In our study, after treatment by DAPT, C13K cells exhibited lower phosphorylation of STAT3 at the serine 727 residue compared with those not treated with DAPT, and in the C13K/si‐Jagged1 cells we found similar results, which is in agreement with the role of p‐STAT3(S727) in chronic lymphocytic leukaemia^43^ and suggested that Jagged1 is an important regulator in the STAT3 signalling pathway. Furthermore, Zhao et al^44^ found STAT3 could direct Jagged1, GDF9 and BMP15 transcription when Rac1 modulates the formation of primordial follicles in mice. By using a co‐IP assay, we also found that STAT3 could physically interact with Jagged1, and in addition we found that EMT key modulator and mesenchymal markers were down‐regulated and epithelial markers were up‐regulated by STAT3 inhibitors and Jagged1 inhibitors, which indicated Jagged1 could crosstalk with the STAT3 pathway and they cooperate to promote the occurrence of EMT in cisplatin‐resistant ovarian cancer cells.

In summary, we defined the mechanism that mediates the crosstalk between Notch and STAT3 pathways in platinum‐resistant ovarian cancer and determined its functional relevance. In this study, we found that STAT3 and Jagged1 are all overexpressed in platinum‐resistant ovarian cancer tissues, and STAT3 is directly regulated by the Notch ligand Jagged1, the leading to aberrant occurrence of EMT, further reinforcing the abilities of invasion and migration of cisplatin‐resistant ovarian cancer cells in vivo and vitro.

## AUTHORS' CONTRIBUTIONS

Jiang Yang: Project development, Data analysis, Manuscript writing; Hui Xing: Fund support; Li Hong: Project development, Manuscript editing; Dan Hua Lu: Project development, Data collection; Bing‐shu Li: Laboratory technique support; Jian‐ming Tang: Data collection; Fengqin Guo: Data management.

## CONFLICT OF INTEREST

The authors have declared that there is no conflict of interest.
