# Supplementary material for: Role of Jagged1/STAT3 signalling in platinum‐resistant ovarian cancer
Source: J Cell Mol Med. 2019 Apr 16;23(6):4005–18. doi: 10.1111/jcmm.14286 (PMC6533470; doi:10.1111/jcmm.14286)
Supplement: Supplementary file 1 [file JCMM-23-4005-s001.doc]

**Supplementary Tables and Figures**

| Table1. The protein expression intensity of tissues in platinum resistant group and platinum responsive group | | | | | | | | | |  | |
| --- | --- | --- | --- | --- | --- | --- | --- | --- | --- | --- | --- |
| Tissue | Total | Notch1（value） | | | *P* | Notch2（value） | | | *P* | |  |
|  |  | ≤2 | 3 | 4 |  | ≤2 | 3 | 4 |  |  |  |
| platinum resistant group | 9 | 0 | 2 | 7 | 0.041 | 0 | 4 | 5 | 0.039 | | |
| platinum responsive group | 33 | 6 | 17 | 10 |  | 10 | 15 | 9 |  |  |  |
| The expression intensity was evaluated by a semiquantitative system to calculate the percentage of positive neoplastic cells: 0 point, no positive cells; 1 point, 1% to 25%; 2 points, 26% to 50%; 3 points, 50% to 75%;4 points, >75%. And ≤2 points judged as negative expression, ≥3 points judged as positive expression. | | | | | | | | | | | |

| Table2. The protein expression intensity of tissues in platinum resistant group and platinum responsive group | | | | | | | | | |  | |
| --- | --- | --- | --- | --- | --- | --- | --- | --- | --- | --- | --- |
| Tissue | Total | E-cadherin（value） | | | *P* | N-cadherin（value） | | | *P* | |  |
|  |  | ≤2 | 3 | 4 |  | ≤2 | 3 | 4 |  |  |  |
| platinum resistant group | 9 | 9 | 0 | 0 | 0.045 | 9 | 4 | 5 | 0.044 | | |
| platinum responsive group | 33 | 18 | 12 | 3 |  | 9 | 18 | 6 |  |  |  |
| The expression intensity was evaluated by a semiquantitative system to calculate the percentage of positive neoplastic cells: 0 point, no positive cells; 1 point, 1% to 25%; 2 points, 26% to 50%; 3 points, 50% to 75%;4 points, >75%. And ≤2 points judged as negative expression, ≥3 points judged as positive expression. | | | | | | | | | | | |

| Table3. The protein expression intensity of tissues in platinum resistant group and platinum responsive group | | | | | | | | | |  | |
| --- | --- | --- | --- | --- | --- | --- | --- | --- | --- | --- | --- |
| Tissue | Total | Vimentin（value） | | | *P* | Twist（value） | | | *P* | |  |
|  |  | ≤2 | 3 | 4 |  | ≤2 | 3 | 44 |  |  |  |
| platinum resistant group | 9 | 1 | 2 | 7 | 0.048 | 0 | 1 | 8 | 0.012 | | |
| platinum responsive group | 33 | 15 | 8 | 10 |  | 7 | 16 | 10 |  |  |  |
| The expression intensity was evaluated by a semiquantitative system to calculate the percentage of positive neoplastic cells: 0 point, no positive cells; 1 point, 1% to 25%; 2 points, 26% to 50%; 3 points, 50% to 75%;4 points, >75%. And ≤2 points judged as negative expression, ≥3 points judged as positive expression. | | | | | | | | | | | |

| Table4. The protein expression intensity of tissues in platinum resistant group and platinum responsive group | | | | | | | | | |  | |
| --- | --- | --- | --- | --- | --- | --- | --- | --- | --- | --- | --- |
| Tissue | Total | STAT3（value） | | | *P* | Jagged1（value） | | | *P* | |  |
|  |  | ≤2 | 3 | 4 |  | ≤2 | 3 | 44 |  |  |  |
| platinum resistant group | 9 | 0 | 0 | 9 | 0.000 | 1 | 3 | 5 | 0.045 | | |
| platinum responsive group | 33 | 5 | 12 | 16 |  | 13 | 15 | 5 |  |  |  |
| The expression intensity was evaluated by a semiquantitative system to calculate the percentage of positive neoplastic cells: 0 point, no positive cells; 1 point, 1% to 25%; 2 points, 26% to 50%; 3 points, 50% to 75%;4 points, >75%. And ≤2 points judged as negative expression, ≥3 points judged as positive expression. | | | | | | | | | | | |


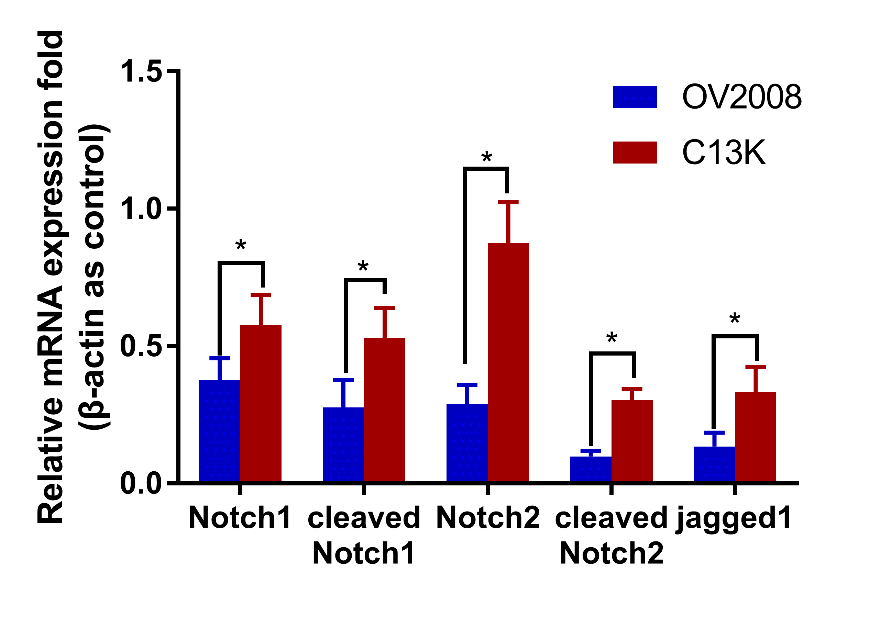


Supplementary Figures1 The mRNA levels of levels of Notch1/2 and cleaved Notch1/2 in OV2008 and C13K cells were examined by qPCR,β-actin was co-amplified as the internal control(*p < 0.05)


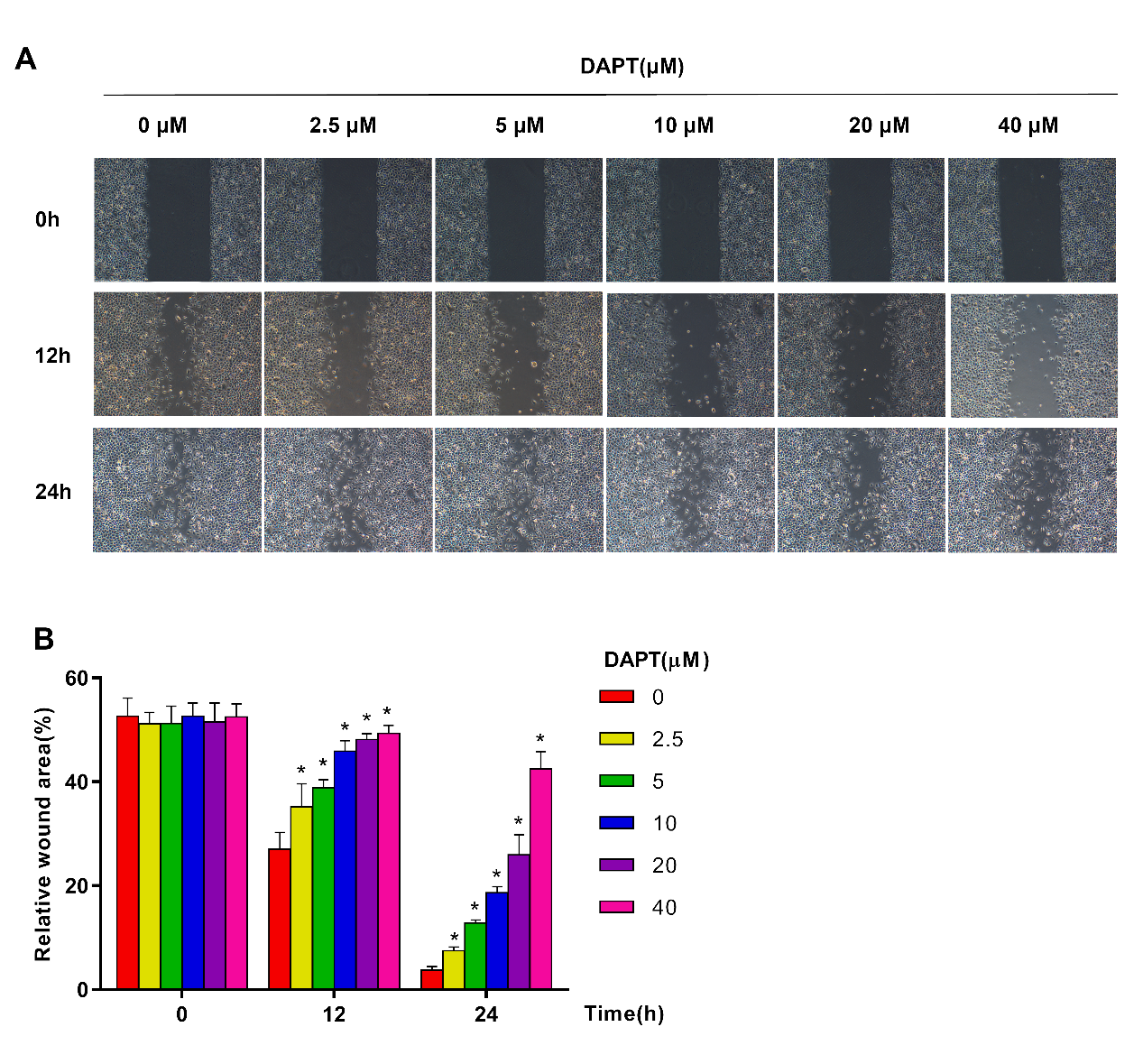
Supplementary Figures2 (A). Wound healing assay was analyzed the migratory ability of C13K cells treated by a wide concentration range of DAPT (0, 2.5, 5,10, 20, 40μM). (B) The quantitative analysis of Supplementary Figures 2A. ( **p* < 0.05)


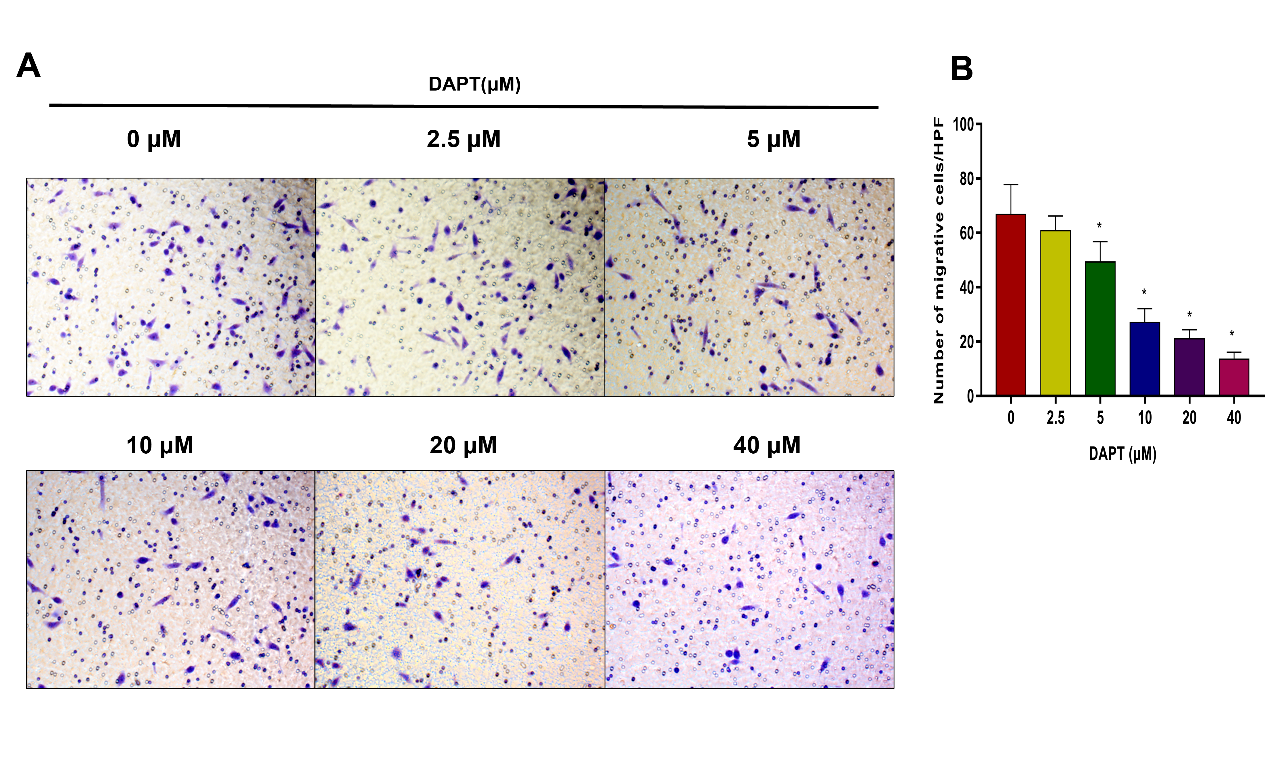
 Supplementary Figures3 (A) Transwell migration assay were performed to confirm the migratory abilities of C13K cells exposed by a wide concentration range of DAPT (0, 2.5, 5,10, 20, 40μM). (B) The quantitative analysis of Supplementary Figures 3A. ( **p* < 0.05)


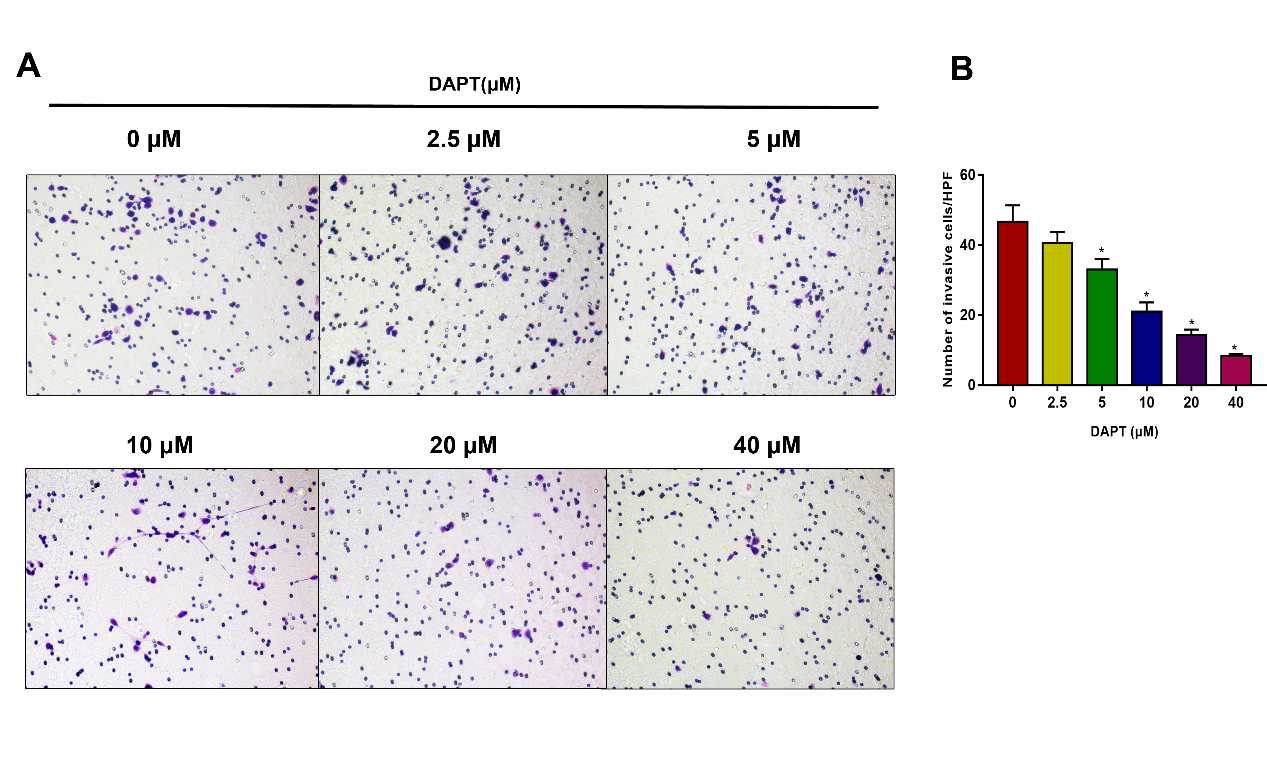
Supplementary Figures4 (A) Transwell migration assay were performed to confirm the invasive abilities of C13K cells exposed by a wide concentration range of DAPT (0, 2.5, 5,10, 20, 40μM). (B) The quantitative analysis of Supplementary Figures4 A. ( **p* < 0.05)


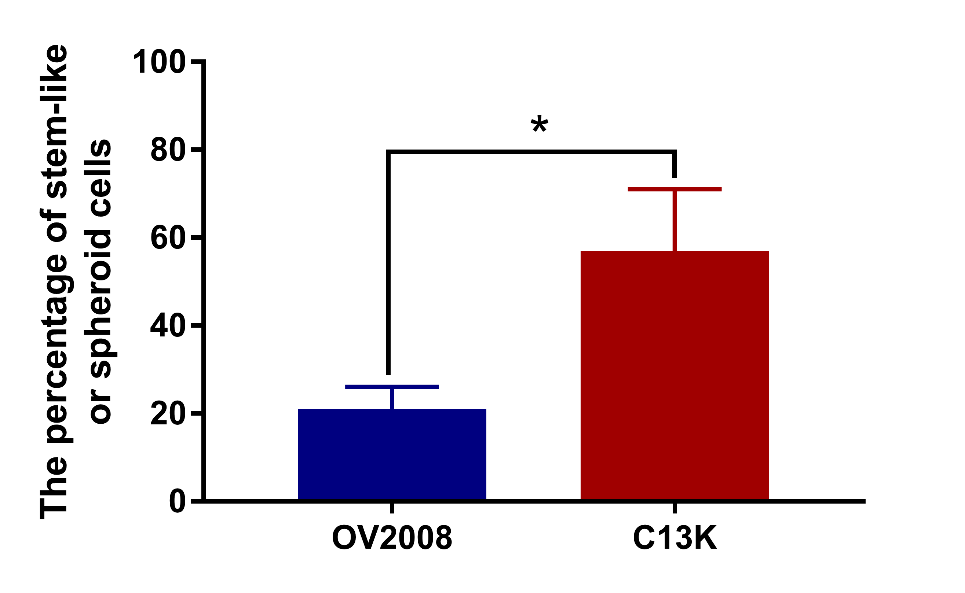


Supplementary Figures 5. the percentage of stem-like or spheroid cells in C13K cells and OV2008 cells, which was designed to observed by Optical microscope. ( *p < 0.05)


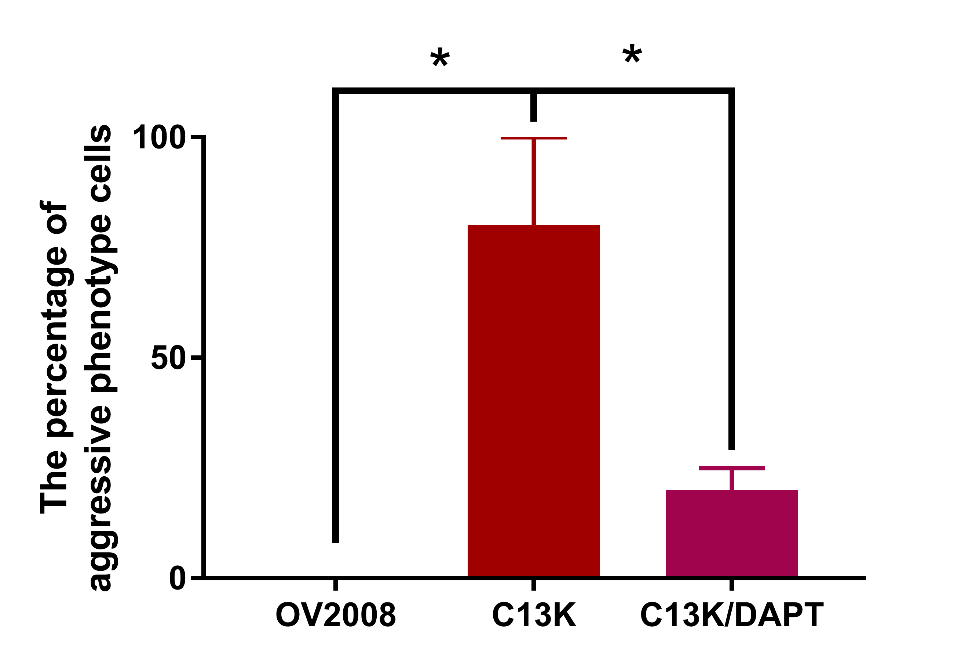


Supplementary Figures 6. the percentage of aggressive phenotype cells in OV2008, C13K cells and C13K/DAPT cells, which was designed to observed by Optical microscope. ( *p < 0.05)


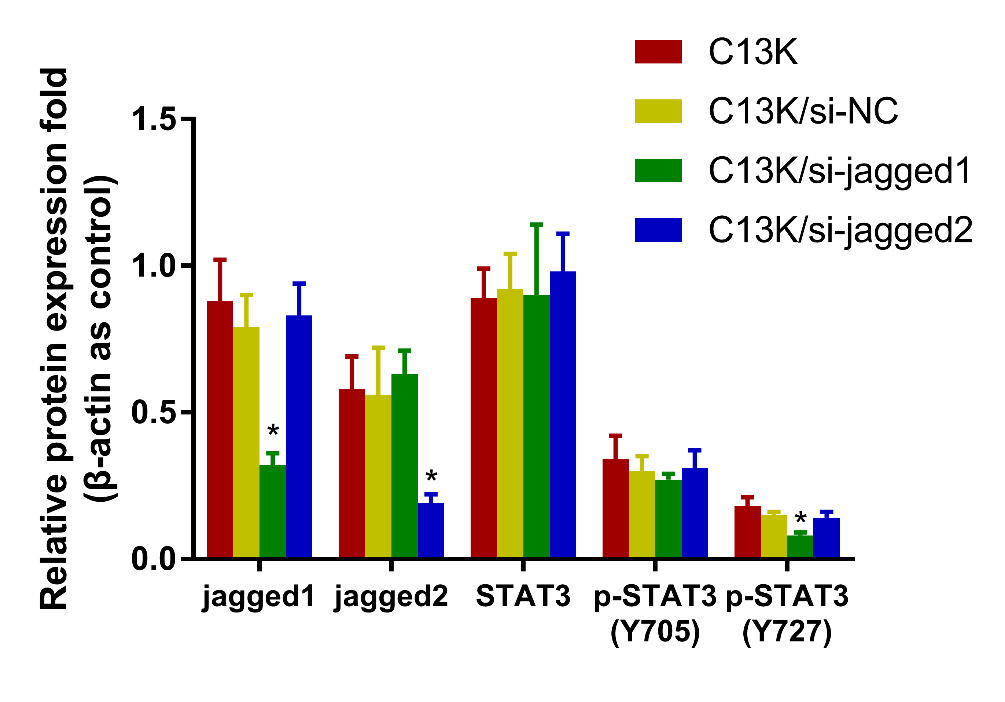
 Supplementary Figures 7. The quantitative analysis of figure 4B, Western blot assay examined the expression of jagged1, jagged2, STAT3, p-STAT3(Y705) and p-STAT3(S727) of C13K cells after transfection with Jagged1 and Jagged2 siRNA. (**p*< 0.05)
